# Validity of the PROMIS^®^ Early Childhood Physical Activity Scale among toddlers

**DOI:** 10.1186/s12966-024-01655-x

**Published:** 2024-09-26

**Authors:** Soyang Kwon, Bridget Armstrong, Nina Wetoska, Selin Capan

**Affiliations:** 1 Buehler Center for Health Policy and Economics, Feinberg School of Medicine, Northwestern University, 750 N Lakeshore Drive, Chicago, IL 60611 USA; 2 Department of Exercise Science, Arnold School of Public Health, University of South Carolina, Columbia, South Carolina USA

**Keywords:** Physical activity questionnaire, ActiGraph accelerometer, Reliability, Young children

## Abstract

**Background:**

The PROMIS^®^ Early Childhood Physical Activity (PROMIS EC PA) scale is a recently developed PROMIS Early Childhood measure to assess PA among children aged 1–5 years. The purpose of this study was to examine test-retest reliability and convergent validity of the PROMIS EC PA scale among toddlers.

**Method:**

An ancillary study was conducted in the toddler-mother dyad sample of the Child and Mother Physical Activity Study. Mothers completed the 7-item PROMIS EC PA scale twice: during a study visit (test) and on the last day when their child’s wore an ActiGraph accelerometer on the hip for 7 days (retest). The PROMIS EC PA summed score was calculated by totaling scores from items 1–5. Test-retest reliability was assessed using intraclass correlation coefficient (ICC) for test and retest PROMIS EC PA. Convergent validity was assessed using rank correlation coefficients (rho) between PROMIS EC PA scores and accelerometer-measured moderate- and vigorous-intensity PA (MVPA).

**Results:**

Among 74 participants (56% female; 19 ± 4 months of mean age with range of 12–30 months), average accelerometer-measured MVPA was 76 ± 24 min/day. The median number of days between PROMIS EC PA test and retest was 8 days (IQR = 6 to 8), with an average PROMIS EC PA summed score of 11.0 ± 3.5 at test and 10.5 ± 3.4 at retest. ICC for the test-retest PROMIS EC PA summed scores was 0.72 (95% CI = 0.59–0.82). The rank correlation between the PROMIS EC PA summed score and accelerometer-measured MVPA was 0.13 (95% CI=-0.10 to 0.35; *p* = 0.28).

**Conclusion:**

In a sample of children aged 12–30 months, test-retest reliability for the PROMIS EC PA scale was moderate and its convergent validity against accelerometer-measured MVPA was poor. Prior to a widespread use of the PROMIS EC PA scale in large-scale research and clinical practice, the tool should be further refined and validated to elucidate how young children’s lived PA experience as measured in the PROMIS EC PA scale is relevant to their health and wellbeing outcomes.

## Introduction

Physical activity (PA) provides various physical and mental health benefits to people of all ages and abilites [1, 2]. It is a global public health concern that many adults and children do not engage in sufficient PA [3], with a longitudinal study [4] suggesting that such physical inactivity habits may be established in early childhood. Recognizing the importance of PA development in early childhood, PA guidelines for young children under 5 years of age have recently been issued by the World Health Organization (WHO) and national public health organizations in Australia, Canada, South Africa and the United Kingdom [5–9]. Correspondingly, increasing research effort has been devoted to assessing PA among young children, particularly using wearable devices, such as accelerometers [10–13]. While accelerometry is useful for determining objective PA, it may not always be feasible or cost effective for large scale monitoring. Thus, a methodological gap exists for a valid tool to assess PA in young children that can be utilized to monitor population-level trends in large-scale surveillances as well as to rapidly evaluate individual PA engagement in clinical practice [14–16]. 

The Patient-Reported Outcome Measurement Information System (PROMIS^®^) Early Childhood (EC) PA scale is a 7-item tool developed by the PROMIS^®^ EC development group to assess PA among children aged 1–5 years. PROMIS EC PA intends to capture children’s lived experiences of PA (e.g., physiological responses of PA, such as sweat or tiredness), which device-based PA assessments fail to capture [17]. The PROMIS EC PA measure is user-friendly and inexpensive to implement [17]. However, as the measure is a relatively new, its reliability and validity have not been tested using empirical data. Rigorous validation of the measure is necessary prior to widespread adoption in research and clinical settings. The aim of this study was to examine test-retest reliability and convergent validity of the PROMIS EC PA scale among toddlers aged 1–2 years. We hypothesized that the test-retest reliability of the PROMIS EC PA scale is good or excellent (an intraclass correlation coefficient [ICC] ≥ 0.75)[18] and convergent validity between PROMIS EC PA score and accelerometer-measured moderate- and vigorous-intensity PA (MVPA) is moderate or higher (rank correlation coefficient rho [ρ] ≥ 0.40) [19]. We additionally explored floor and ceiling effects, hypothesizing that a low proportion of the sample with the lowest possible (“floor”) and highest possible (“ceiling”) PROMIS EC PA scores are 15% or less [20]. 

## Methods

An ancillary study was conducted to test the validity of the PROMIS EC PA scale in a toddler-mother dyad sample from the Child and Mother Physical Activity Study (CAMPAS). CAMPAS is an ongoing longitudinal study that investigates PA development from age 1 to 3 years [21]. The eligibility criteria for child participants included being age 10 to 15 months at baseline assessment, having no cerebral palsy or other medical conditions precluding physical movement, and residing in the Chicago metropolitan area. The eligibility criteria for mother participants included self-identifying as the mother of the participating child, being 18 years or older, living with the child at least 50% of the time, and speaking English or Spanish. Recruitment was performed via flyers distributed to various community locations and via email blasts to potentially eligible participants extracted from a single healthcare system’s electronic patient database. CAMPAS performed in-person and remote assessments longitudinally six months apart. Detailed information about CAMPAS can be found in our prior publication [21]. Between December 2023 and April 2024, CAMPAS participants who had any waves of CAMPAS assessments were asked to complete the PROMIS EC PA scale for the ancillary study.

### Measurements

**Demographics**,** growth**,** and development.** Participants’ mothers completed an online demographic survey that asked about child sex, age, racial/ethnic background as well as maternal education and residential address. Mothers also reported whether the participating child was able to walk independently. Residential address was used to assess neighborhood resources based on the Child Opportunity Index (COI) [22]. Each participant was assigned to one of the five Chicago metropolitan COI categories: very low, low, moderate, high or very high. Mothers provided a copy of the most recent child clinic visit summary that contained a date of visit and length and weight measurements. WHO weight-for-length percentile [23] was calculated based on the clinic anthropometry measurements, which was then dichotomized into < 85 and ≥ 85th percentile [24]. 

**PROMIS Early Childhood Physical Activity Scale.** The PROMIS EC PA Parent-Report Scale v1.0 is a 7-item scale developed by applying the PROMIS methodology standards [17]. The 7 items ask about activities in the past 7 days, with an emphasis on capturing children’s lived experience of PA [11]. Question items are listed in Table 1. During a study wave, mothers were asked to complete the PROMIS EC PA measure twice, approximately 8 days apart; as a part of the CAMPAS online survey (“test”) as well as in a paper form on the last day of a child’s 7-day accelerometer wear (“retest”). Item responses were scored in accordance with the scoring manual: 1 = no days, 2 = 1 day, 3 = 2–3 days, 4 = 4–5 days, and 5 = 6–7 days for items 1 to 6 and 1 = not at all, 2 = a little bit, 3 = somewhat, 4 = quite a bit, 5 = very much for item 7. Items 1–5 were used to create a summed score. Items 6 and 7 were individually scored [17]. 

**Table 1:** Descriptive statistics for the PROMIS early childhood physical activity scale among children aged 1–2 years

	Test (*n* = 72)				Retest (*n* = 74)			
Item	Mean ± SD	Median (IQR)	Floor,^a^ %	Ceiling,^a^ %	Mean ± SD	Median (IQR)	Floor,^a^ %	Ceiling,^a^ %
1. How many days did your child so physically active that he/she sweated?	1.9 ± 1.3	1 (1, 3)	58	6	1.8 ± 1.0	1 (1, 3)	54	1
2. How many days did your child play so hard that he/she got physically exhausted?	2.4 ± 1.2	2.5 (1, 3)	31	4	2.4 ± 1.1	3 (1, 3)	32	1
3. How many days did your child play so hard that he/she fell asleep early?	1.9 ± 0.9	2 (1, 3)	42	0	1.7 ± 0.9	1 (1, 2)	51	0
4. How many days did your child play so hard that he/she needed an extra or longer nap?	2.0 ± 0.9	2 (1, 3)	43	0	1.9 ± 0.9	2 (1, 3)	42	1
5. How many days did your child play so hard that he/she felt tired?	2.7 ± 1.4	3 (2, 3)	22	10	2.6 ± 1.2	2 (1, 3)	22	7
6. How many days did your child do vigorous physical activities for 30 min or more?	2.7 ± 1.4	3 (1, 4)	29	13	3.0 ± 1.4	3 (2, 4)	22	16
7. On a usual day, how physically active was your child?	4.2 ± 0.8	4 (4, 5)	0	35	3.9 ± 0.7	4 (4, 4)	0	18
PROMIS EC PA items 1–5 summed score	10.9 ± 3.5	11.5 (8.5, 13.0)	11	0	10.4 ± 3.4	10 (9, 13)	14	0

^a^“Floor” refers to percentage of participants who endorsed “no days” or “not at all”; “ceiling” refers to percentage of participants who endorsed “6–7 days” or “very much”

IQR, interquartile range; M ± SD, mean ± standard deviation; PROMIS EC PA, PROMIS^®^ Early Childhood Physical Activity

**Accelerometer assessment.** We used ActiGraph GT3X-BT accelerometers (ActiGraph LLC; Pensacola, FL). During an in-person or virtual study visit, mothers were given instructions on accelerometer wear. Mothers received an accelerometer package that contained an accelerometer with an adjustable waist belt, an instruction sheet, a wear log sheet, a hard copy of the PROMIS EC PA scale, and a prepaid return envelope during an in-person visit or via mail. Mothers were asked to assist their child’s in wearing an accelerometer on the hip for 7 days and 24 h. Upon completion of the 7-day wear, the package was returned via mail. Participants who wore the accelerometer for 3 days or less were asked to complete a re-wear.

Accelerometer data was downloaded and reintegrated in 15-second epochs using the ActiLife software version 6.13. Accelerometer data collected between 6 AM and 10 PM were extracted [25–27]. Non-wear periods, defined as periods with ≥ 20 consecutive zero counts [28–31], were excluded. Then, valid wear days (≥ 8 wear hours/day between 6 AM and 10 PM) were selected [28, 32, 33]. For each valid day, we calculated minutes spent in MVPA, which was defined as > 417 counts per 15 s [10, 30]. Average daily minutes spent in MVPA (minutes/day) was calculated per child.

### Statistical analysis

All statistical analyses were conducted using SAS 9.4 (Cary, NC). Descriptive analyses were performed for all study variables. Cronbach’s alpha was calculated to measure the internal consistency of PROMIS EC PA items 1-5 [34, 35]. To examine test-retest reliability, we calculated ICC (moderate if ICC = 0.50–0.74; good if ICC = 0.75–0.89; excellent if ICC ≥ 0.90[18]) between two repeated measures of the PROMIS EC PA scale among participants who completed the tool twice within 14 days, using the SAS ICC9 marco [36]. 

To examine floor and ceiling effects, we calculated the proportion of participants who endorsed “no days” or “not at all” (“floor”) and the proportion of participants who endorsed “6–7 days” or “very much” (“ceiling) in PROMIS EC PA question items [17]. We also calculated the proportion of participants with a PROMIS EC PA summed score of 5 (“floor”) and 25 (“ceiling”).

To examine convergent validity, we calculated Spearman correlation coefficients (ρ) between the retest PROMIS EC PA score and accelerometer-measured MVPA among participants who completed both retest PROMIS EC PA and accelerometer assessment (negligible correlation if ρ = 0.00-0.09; weak correlation if ρ = 0.10–0.39; moderate correlation if ρ = 0.40–0.69; strong correlation if ρ = 0.70–0.89; very strong correlation if ρ = 0.90-1.00).[19] We used the retest data to align the accelerometer wear period and the PROMIS EC PA’s 7-day recall period. These analyses were repeated separately by sex, age group (1 year vs. 2 years), and ability to walk independently (yes vs. no) to explore whether test-retest reliability and convergent validity differ by sex, age group, and walking ability.

### Power consideration

Power calculation for test-retest reliability was performed to detect a good or excellent reliability (ICC ≥ 0.75), under the alternative hypothesis of moderate reliability (ICC = 0.50) [18]. Power calculation indicated that a sample size of 36 provides 80% power to detect ICC ≥ 0.75 (null ICC = 0.50) using F-test at a significance level of 0.05 (two-sided). In power calculation for convergent validity, we assumed that the rank correlation coefficient (ρ) between a PROMIS EC PA summed score and accelerometer-measured MVPA would be moderate or higher (ρ ≥ 0.40),[19] as prior convergent validity studies among children reported correlation levels of 0.35-41 between a PA questionnaire (PAQ) and accelerometer-measured PA [37–39]. Power calculation indicated that a sample size of 51 provides 80% power to detect ρ ≥ 0.40, under the null hypothesis ρ = 0.00 at a significance level of 0.05 (two-sided).

## Results

A total of 74 participants (41 females;52%) participated in the ancillary study. Average age was 19 ± 4 months with a range of 12–30 months. Only 8 of 74 children (11%) were 2 years old. Of the 74, 39 (53%) were non-Hispanic white, 14 (19%) Hispanic, 9 (12%) non-Hispanic Black, 9 (12%) non-Hispanic multi-race, and 3 (4%) non-Hispanic Asian or Middle Eastern; 24% resided in a neighborhood with very low or low COI; 88% had mothers with a 4-year college degree or higher education; and 29% had WHO weight-for-length above the 85th percentile [23]. All participants had at least 4 valid accelerometer days (range of 4–7 days; median of 7 days). Average valid wear was 14.7 ± 1.5 h/day. Average accelerometer-measured MVPA was 76 ± 24 min/day.

**Reliability.** Reliability was examined among 65 participants, after excluding 7 participant who completed the test and retest more than 14 days apart and 2 participants who completed the PROMIS EC PA scale only once. The number of days between test and retest ranged from 5 to 14 days (median of 8 days; interquartile range of 6–8 days).

Cronbach’s alpha of the PROMIS EC PA items 1–5 was 0.63 at test and 0.67 at retest. ICC for the PROMIS EC PA summed score was 0.72 (95% confidence interval [CI] = 0.59–0.82; Table 2). The ICC did not significantly differ by sex, age, or ability to walk independently (Table 2). ICCs for PROMIS EC item 6 and 7 scores were 0.63 (95% CI = 0.48–0.75) and 0.58 (95% CI = 0.42–0.72), respectively.

**Table 2:** Accelerometer-measured moderate and vigorous-intensity physical activity (MVPA) and PROMIS^®^ early childhood physical activity summed scores among 65 children aged 1–2 years

Total	Sample, *n* (%)	Accelerometer-measured MVPA minutes/day, M ± SD	PROMIS EC PA summed score at test, M ± SD	PROMIS EC PA summed score at retest, M ± SD	ICC for PROMIS EC PA test-retest reliability (95% CI)
Total	65	77 ± 24	11.0 ± 3.5	10.5 ± 3.4	0.72 (0.59, 0.82)
Sex					
Male	30	80 ± 25	11.3 ± 3.6	10.9 ± 3.6	0.70 (0.49, 0.85)
Female	35	75 ± 23	10.7 ± 3.5	10.1 ± 3.2	0.73 (0.56, 0.86)
Age					
12–23 months	57	72 ± 23	10.9 ± 3.6	10.3 ± 3.4	0.71 (0.56, 0.82)
24–30 months	8	87 ± 33	11.8 ± 3.4	11.4 ± 3.3	0.79 (0.45, 0.95)
Ability to walk independently					
Yes	46	80 ± 23	11.0 ± 3.7	10.8 ± 3.3	0.72 (0.56, 0.84)
No	19	69 ± 27	10.8 ± 3.3	9.6 ± 3.5	0.70 (0.44, 0.88)

CI, confidence interval; ICC, intraclass correlation coefficient; M ± SD, mean ± standard deviation; MVPA, moderate- and vigorous-intensity physical activity; PA, physical activity; PROMIS EC PA, PROMIS^®^ Early Childhood Physical Activity

**Fig. 1:**
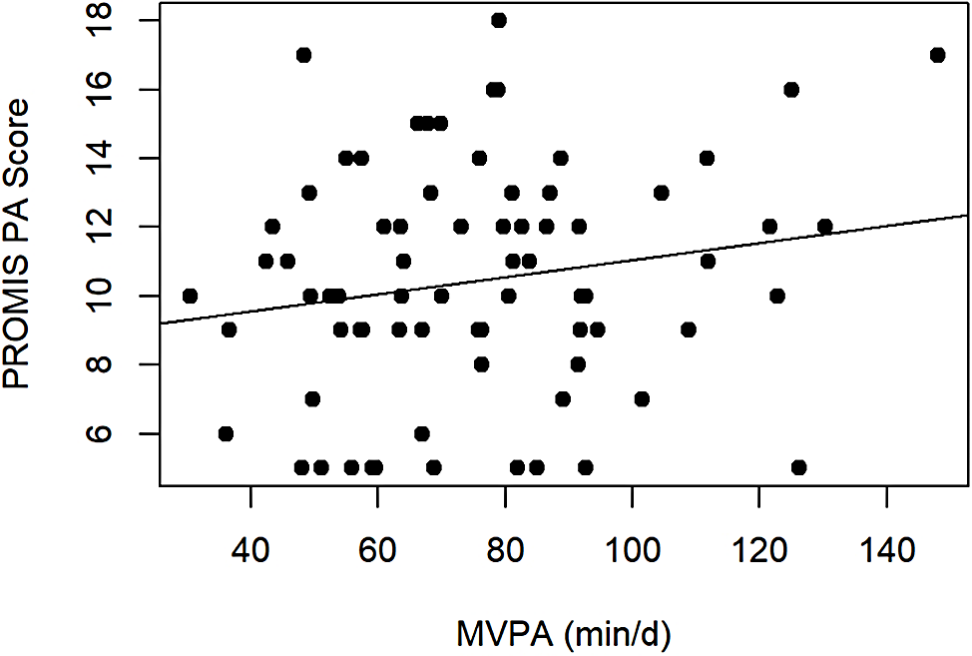
Scatter plot and a line of best fit for PROMIS^®^ Early Childhood Physical Activity (PROMIS EC PA) summed scores over accelerometer-measured moderate and vigorous-intensity physical activity (MVPA)

**Validity.** Validity was examined among 74 participants who completed the PROMIS EC PA retest and accelerometer wear. In floor and ceiling effect examination, the proportion of participants with the lowest and highest possible PROMIS EC PA summed scores were 14% and 0%, respectively, at retest (Table 1). In convergent validity examination, we found no significant linear correlation between the retest PROMIS EC summed score and accelerometer-measured MVPA (ρ = 0.13 [95% CI=-0.10 to 0.35]; *p* = 0.28; Fig. 1). In sex-specific analysis, Spearman correlation coefficients were 0.03 (*p* = 0.83) for females and 0.31 (*p* = 0.08) for males. In age group-specific analysis, Spearman correlation coefficients were 0.11 (*p* = 0.39) for children aged 12–23 months and 0.56 (*p* = 0.15) for children aged 24–30 months. In subgroup analysis by ability to walk independently, Spearman correlation coefficients were 0.15 (*p* = 0.29) for children who could walk independently and 0.17 (*p* = 0.45) for children who could not walk independently. Spearman correlation coefficients between retest PROMIS EC item 6 and 7 scores and accelerometer-measured MVPA were 0.02 (*p* = 0.89) and 0.17 (*p* = 0.14), respectively.

## Discussion

This study found that test-retest reliability for the PROMIS EC PA summed score (items 1–5) was estimated moderate (ICC = 0.72)[18] among children aged 12–30 months. Test-retest reliability for the PROMIS EC PA items 6 and 7 was also estimated moderate [18] (ICC = 0.63 and 0.58, respectively). The internal consistency of the PROMIS EC PA items 1 to 5 (Cronbach’s alpha = 0.63–0.67) was below an acceptable level [34, 35]. The convergent validity of the PROMIS EC PA against accelerometer-measured MVPA was found to be poor with a weak correlation (ρ = 0.13) [19]. Potential floor effects (14% with the lowest possible score) [20] were present in this sample.

This is one of the first studies to examine the validity of the PROMIS EC PA scale using empirical data. Internal consistency for the PROMIS EC PA items 1–5 was found to be unacceptably lower (Cronbach’s alpha = 0.63–0.67) in this study sample than shown in the tool development process (Cronbach’s alpha = 0.82) [17]. The test-retest reliability was evaluated below good (ICC = 0.72). This test-retest reliability level is inferior to that of the Movement Behavior Questionnaire-Child (MBQ-C) PA scale that was implemented 3 days apart among children aged 18 month to 5 years (ICC = 0.80–0.88), while it was superior to that of the lengthy Early Years PAQ that was implemented 7 days apart on average among children aged 18 months to 4 years (ICC = 0.35) [40]. In this sample, although it was not statistically significant, the ICC was slightly higher among 2-year-old children (ICC = 0.79; *n* = 8) than 1-year old children (ICC = 0.71; *n* = 57). We evaluate that the reliability of PROMIS EC PA is considered below acceptable among children aged 12–30 months, while it should be further evaluated in a larger sample of 2-year-old children.

We found that the convergent validity of PROMIS EC PA against accelerometer-measured MVPA was poor among children aged 12–30 months, indicated by a weak correlation (ρ = 0.13) [19]. This correlation is much lower than previously reported correlation levels between parent-reported PA and sensor-measured PA among young children: rank correlation coefficients of 0.35–0.39 between the MBQ-C PA energetic play and accelerometer-measured MVPA among Australian children aged 18 months to 5 years; [39] a rank correlation coefficient of 0.39 between Canadian Health Measures PAQ and accelerometer-measured total PA among Canadian children under age 6 years; [41] a rank correlation coefficient of 0.30 between the Early Years PAQ and accelerometer-measured MVPA among British children aged 18 months to 4 years; [40] and rank correlation coefficients of 0.33–0.39 between an outdoor playtime recall questionnaire and accelerometer-measured MVPA among American and Brazilian preschool-aged children [42, 43]. It was also lower than a Pearson correlation coefficient of 0.35 between the PROMIS Pediatric PA scale and Fitbit-measured daily step counts among adolescents [38]. Given our subgroup analysis suggested higher convergent validity among males (ρ = 0.31) and among 2-year-old children (ρ = 0.56), the convergent validity of the PROMIS EC PA scale may be higher among more active young children.

Poor convergent validity against an accelerometer does not directly imply that the PROMIS EC PA is not valid, per se. Unlike criterion validity that is assessed by comparing a testing measure against a criterion measure (i.e., gold standard), convergent validity compares a testing measure against a non-criterion reference measure. Accelerometer-measured MVPA is widely accepted as an objective measure of PA level among children [44, 45]. Therefore, the poor convergent validity result suggests that PROMIS EC PA may not be valid as a measure of PA level; however, it is still possible that PROMIS EC PA measures another PA dimension (e.g., physiological symptoms of PA), as the tool is intended to measure lived experiences of PA.

Prior studies attempted to explain the low validity of self- or parent-reported PAQs for children. Marasso et al. [46] interpreted that the difference between a PAQ score and accelerometer-measured MVPA among children could reflect the reporter’s difficulty in judging and feeling one’s physical engagement. In understanding how parents retrieve and formulate response to PAQ for their child, Byrne et al. [47] reported that parents thought about their child’s daily routine (e.g., outdoor time, wake and bedtime) regardless of the actual intensity of PA engaged. Singh et al. [48] discussed that no differences in the PROMIS Pediatric PA scores between two groups of children with sickle cell disease that are known to have different levels of PA could be because the PROMIS Pediatric PA scale is simply not valid to measure PA level. All could partly explain our low convergent validity finding. In addition, the young age range of this study sample could factor into the low convergent validity: for toddlers who have just begun to walk independently, questions about “sweating” due to PA (item 1) and playing so hard to get physically “exhausted” (item 2) could be less relevant compared to preschool-aged children (3–5 years of age). Our study sample was relatively active, engaging in MVPA for 76 min on average (compared to 60 min/day in a meta-analysis [49]), which was also indicated by PROMIS EC PA item 7 where 78% of the sample reported that their child was physically active quite a bit or very much on a usual day at retest. In contrast, 54% reported “*no days*” to the question about how many days their child was physically active that he/she sweated (item 1), 32% reported “*no days*” to the question about how many days their child played so hard that he/she got physically exhausted (item 2), and 51% reported “*no days*” to the question about how many days their child played so hard that he/she fell asleep early (item 3). These proportions were much higher than shown in the tool development (21% for item 1; 27% for item 2; and 15% for item 3[17]). Conversely, the proportion for the “ceiling” response (“6–7 days”) in the present study was lower than shown in the tool development [17]. Future research should investigate whether physiological symptoms (e.g., “sweat,” “exhausted”) assessed in the PROMIS EC PA measure are appropriate to children at toddler age.

Given that an accelerometer is widely accepted as an objective measure of PA level in examining the impacts of PA on health and wellbeing outcomes among young children [50–52], our poor convergent validity result for the PROMIS EC PA against accelerometer-measured MVPA raises questions on whether and how the PROMIS EC PA scale can be useful and how any relationships of health outcomes with PROMIS EC PA scores, which could be drastically different from the relationships with accelerometer-measured PA metrics, should be interpreted in future large-scale research studies. If found to be valid and reliable, the PROMIS EC PA scale has a potentially great significance in that it enhances continuity of well-known PROMIS across the lifespan by filling the PROMIS assessment void for young children, and it is a user-friendly and inexpensive tool to assess PA among young children in large-scale research and clinical practice. However, prior to a widespread use of the PROMIS EC PA in large-scale research and clinical practice, the tool should be further validated to elucidate how young children’s lived PA experience as measured in the PROMIS EC PA is pertinent to their health and wellbeing outcomes.

Limitations of the current study should be acknowledged. Because the study sample was relatively active, the results may not be generalizable to toddler populations with low PA. The study results also cannot be generalized to children aged 3–5 years, because this study only included a sample of children aged 12–30 months.

## Conclusions

This study found that test-retest reliability for the PROMIS EC PA measure was moderate and the convergent validity of the PROMIS EC PA against accelerometer-measured MVPA was poor among children aged 12–30 months. Prior to a widespread use of the PROMIS EC PA in large-scale research studies and clinical practices, the measure should be further validated to elucidate how children’s lived PA experience as measured in the PROMIS EC PA is relevant to health and wellbeing outcomes among young children.

## Data Availability

Data may be available upon request.

## Abbreviations

CI: Confidence interval
ICC: Intraclass correlation coefficient
MVPA: Moderate- and vigorous-intensity physical activity
PA: Physical activity
PAQ: Physical activity questionnaire
PROMIS EC PA: Patient-Reported Outcome Measurement Information System Early Childhood Physical Activity
